# Evaluation of Structurally Different Ionic Liquid-Based Surfactants in a Green Microwave-Assisted Extraction for the Flavonoids Profile Determination of *Mangifera* sp. and *Passiflora* sp. Leaves from Canary Islands

**DOI:** 10.3390/molecules25204734

**Published:** 2020-10-15

**Authors:** Kristýna Moučková, Idaira Pacheco-Fernández, Juan H. Ayala, Petra Bajerová, Verónica Pino

**Affiliations:** 1 Department of Analytical chemistry, Faculty of Chemical Technology, University of Pardubice, Studentská 573, 53210 Pardubice, Czech Republic; kristyna.mouckova@student.upce.cz (K.M.); petra.bajerova@upce.cz (P.B.); 2 Laboratorio de Materiales para Análisis Químicos (MAT4LL), Departamento de Química, Unidad Departamental de Química Analítica, Universidad de La Laguna (ULL), 38206 Tenerife, Spain; jayala@ull.edu.es; 3 Instituto Universitario de Enfermedades Tropicales y Salud Pública de Canarias, Universidad de La Laguna (ULL), 38206 Tenerife, Spain

**Keywords:** ionic liquid-based surfactant, microwave-assisted extraction, mango, passion fruit, bioactive compounds, flavonoids, plant by-products, green extraction

## Abstract

Aqueous solutions of ionic liquids (ILs) with surface active properties were used as extraction solvents, taking advantage of their impressive solvation properties, in a green microwave-assisted solid-liquid extraction method (IL-MA-SLE) for the extraction of flavonoids from passion fruit and mango leaves. The extraction method was combined with high-performance liquid chromatography and photodiode-array detection (HPLC-PDA) and optimized by response surface methodology using the Box-Behnken experimental design. Under optimum conditions, the extraction efficiency of six structurally different IL-based surfactants was evaluated. Thus, imidazolium-, guanidinium- and pyridinium-type ILs with different tailorable characteristics, such as side chain length and multicationic core, were assessed. The decylguanidinium chloride ([C_10_Gu^+^][Cl^–^]) IL-based surfactant was selected as key material given its superior performance and its low cytotoxicity, for the determination of flavonoids of several samples of *Passiflora* sp. and *Mangifera* sp. leaves from the Canary Islands, and using as target analytes: rutin, quercetin and apigenin. The analysis of 50 mg of plant material only required 525 µL of the low cytotoxic IL-based surfactant solution at 930 mM, 10.5 min of microwave irradiation at 30 °C and 50 W, which involves a simpler, faster, more efficient and greener method in comparison with other strategies reported in the literature for obtaining bioactive compounds profiles from plants.

## 1. Introduction

Nowadays, much attention is paid to the incorporation of the Green Chemistry principles in analytical extraction methods [1]. One of the main strategies is the use of new solvents to replace conventional organic solvents, which are characterized by their high volatility, flammability and toxicity. In this sense, ionic liquids (ILs) constitute a group of nonmolecular solvents with melting points below 100 °C, prepared by the combination of bulky organic cations and organic or inorganic anions. Depending on the number of cation moieties, they divide into two main groups: monocationic and multicationic ILs [2,3]. They have gained considerable popularity in analytical sample preparation in recent years for presenting a number of outstanding features for solvent extraction processes, such as great solvation ability, high thermal, chemical and electrochemical stability, nonflammability, and negligible volatility at room temperature, which confers them low toxicity in comparison with conventional solvents [2]. Most importantly, ILs are easily tunable by selecting the adequate combination of cation and anion, or by incorporating different functional groups in their structures. This allows the preparation of ILs with specific properties, since even a simple modification in the IL structure leads to significant changes in their properties. Given this impressive versatility, a wide range of ILs derivates have been described, including IL-based surfactants [3,4,5,6,7].

IL-based surfactants are ILs capable of forming micellar aggregates when dissolved in water above a certain concentration, known as a critical micellar concentration (CMC) [8]. Therefore, they present the inherent properties of ILs but with improved solvation properties due to the formation of micelles. In general, they present CMC values lower than conventional surfactants with similar structures [8]. Moreover, multicationic IL-based surfactants present even lower CMC in comparison with their analogue monocationic IL-based surfactants and subsequently, much lower amounts of IL-based surfactants can be used to take advantage of their surface-active properties [8]. Considering this interesting set of characteristics, there is an increasing number of applications of IL-based surfactants in analytical extraction strategies [3]. Particularly, aqueous solutions of IL-based surfactants have been explored as a promising alternative to organic solvents for the extraction of bioactive compounds from plant materials in solid–liquid extraction methods (SLE) [4,5,6,7]. The solvation characteristics of IL-based surfactants allow the extraction of a wide variety of compounds from the plants, with diverse polarity and characteristics, thus aiding in the determination of their composition profile. This type of analysis is especially useful for the valorization of plants’ by-products [9].

Indeed, the growing demand in valorization of plants’ by-products (i.e., leaves, peels, seeds, pulps), with the aim of using them as a renewable source of bioactive compounds for numerous applications within the pharmaceutical, cosmetic and food industries [10], is also impelled by the Green Chemistry trend. These by-products represent a disposal problem for agricultural and food industry, and their revaluation is of special interest. These agri-food by-products are rich in natural bioactive compounds such as flavonoids, carotenoids and phytosterols, among others [11,12]. For example, flavonoids are secondary metabolites, which are widely spread in the plants and are known mainly for their antioxidant capacity. They are capable of neutralizing free-radicals responsible for cell damage [13].

*Mangifera* sp. (Anacardiaceae) and *Passiflora* sp. (Passifloraceae), which are one of the most cultivated tropical fruits in the Canary Islands, are very interesting for the valorization of their by-products because they are not effectively utilized and present a high content of polyphenols. Besides its most popular use as a delicate fruit, several species from *Passiflora* genus have a history of use in a traditional herbal medicine [14]. In general, flavonoids and flavonoid glycosides are present in high amounts in most of the *Passiflora* species [15]. Some of them have been previously examined, including isoorientin, orientin, isovitexin or vitexin [16]. Mango is also a popular tropical fruit known for its great nutritional composition and beneficial health properties, such as antioxidant, antiproliferative or anti-inflammatory activities. *Mangifera* sp. contains various classes of polyphenols, carotenoids and ascorbic acid [17,18]. In the case of both fruits, their beneficial and antioxidant properties are attributed to their secondary metabolites, namely the polyphenolic compounds. Therefore, the by-products of passion fruit and mango could be used as a natural source of antioxidants or as functional food additives, therefore improving the environmental impact of food by-products waste [13,19] and clearly generating an added economical value.

Mastellone et al. recently developed a microwave-assisted SLE method using an imidazolium IL-based surfactant (IL-MA-SLE) for the determination of the polyphenolic profile of *Vitis vinifera* leaves [20]. The study demonstrated the superior performance of the method in terms of greenness, speed and effectiveness, particularly in comparison with the conventional UA-SLE method that uses methanol as extraction media [20]. Despite the success of the method, it is important to highlight current trends to ensure the design of IL with negligible toxicity, given the risks associated to the most used imidazolium-based ILs. In this sense, ILs with fluorine-free anions and with more biodegradable cations (e.g., pyridinium or guanidinium) are more advisable when tailoring ILs for targeted extractions. Moreover, the longer the IL side chain in any of the moieties, the more toxic the resulting IL [2,21].

Therefore, the aim of this study is to evaluate the influence of the IL structure in a MA-SLE extraction method of flavonoids from plant leaves, intending significant improvements in the greenness of the entire method compared to previous IL-based approaches [18]. Thus, six ILs-based surfactants are assessed, containing different cation moieties (imidazolium, guanidinium and pyridinium) and with different structural characteristics (monocationic versus multicationic or with different alkyl chain lengths). The method integrates high-performance liquid chromatography and photodiode array detector (HPLC-PDA) for determining three target flavonoids (rutin, quercetin and apigenin) in both *Passiflora* sp. and *Mangifera* sp. leaves. The method was thoroughly optimized using the Box-Behnken experimental design, and the optimum IL-based surfactant was used to determine flavonoids profiles in several lines and cultivars of the selected plant leaves.

## 2. Materials and Methods

### 2.1. Chemicals, Reagents and Samples

Hexadecylpyridinium bromide IL ([C_16_Py^+^][Br^–^]) (97%) was purchased from Sigma-Aldrich (Steinheim, Germany). The guanilating agent 1H-pyrazole-1-carboxamidehydrochloride (99%), decylamine (99%) and octylamine (99%), all used to synthesize guanidinium ILs, were also purchased from Sigma-Aldrich. For the synthesis of imidazolium ILs, 1-methylimidazole, 1-butylimidazole, 1-bromohexadecane, imidazole, 1-bromobutane, 1-bromooctane and dimethylsulfoxide, were supplied by Sigma–Aldrich (St. Louis, MO, USA). Isopropanol, ethyl acetate, dichloromethane, chloroform, potassium hydroxide, acetonitrile, 1,3,5-tris(bromomethyl)benzene and diethyl ether, were acquired from Fisher Scientific (Fair Lawn, NJ, USA). Ethanol LiChrosolv^®^ grade LC was supplied by Merck KGaA (Darmstadt, Germany), while methanol Chromasolv^TM^ grade LC was purchased from Honeywell (Seelze, Germany).

The studied analytes belong to the group of flavonoids, including rutin (95%), quercetin (95%) and apigenin (95%), and were all supplied by Sigma-Aldrich. Individual standard solutions were prepared in acetonitrile HiPerSolv Chromasolv^TM^ grade LC supplied by VWR (Llinars del Vallés, Spain) and at the following concentrations: 500 mg·L^−1^ for rutin, 804 mg·L^−1^ for quercetin, 500 mg·L^−1^ for apigenin. Working standard solutions containing the target analytes were prepared at concentrations ranging from 0.03 to 500 mg·L^−1^ in acetonitrile. All standard solutions were kept protected from light at 4 °C.

Ultrapure water with 18.2 MΩ·cm was obtained from a Milli-Q water purification system (Millipore, Bedford, MA, USA). Acetonitrile HiPerSolv Chromasolv^TM^ grade LC supplied by Honeywell Riedel-de Haën™ (Seelze, Germany), and acetic acid (99%) supplied by Sigma-Aldrich, were used in the chromatographic separation.

Leaves of 5 different seed-propagated lines of *Passiflora* sp. and 4 different cultivars of *Mangifera* sp. were supplied by the Canarian Institute of Agricultural Research (Valle de Guerra, Tenerife, Spain). Table S1 of the Supplementary Material (SM) includes the identification of the samples, together with images of the leaf anatomy. The plants of the same species were grown in the sample plot, and the leaves were collected when they were in the same physiological state in November 2019. All leaves were lyophilized, grinded into powder and stored in plastic sampling containers, which were placed in a dryer.

### 2.2. Material, Instrumentation and Equipment

A Sartorius analytical balance (Madrid, Spain) with a minimum readability of 0.1 mg was used. Hot-plate magnetic stirrers and a RV 10 digital rotary evaporator with temperature control from IKA^®^ (Staufen, Germany), which was equipped with a vacuum pump VP 2 Autoyac from Vacuubrand (Wertheim, Germany), were used in the synthesis of the ILs. Synthesized ILs were characterized by ^1^H-NMR using an AVANCE™ NMR spectrometer (500 MHz) from Bruker (Billerica, MA, USA).

PYREX ^®^ (Staffordshire, UK) centrifuge tubes of 25 mL (10 cm length × 2.6 cm outer diameter), magnetic stir bars (12.7 × 3.2 mm) from Sigma-Aldrich, a focused microwave synthesizer Discover^®^ SP by CEM (Matthews, NC, USA), a 5702 centrifuge from Eppendorf, and an ultrasonic bath KM by Shenzhen Codyson Electrical Co., Ltd. (Shenzhen, China), were used to perform the extraction procedures. A 2 mL glass syringe Fortuna Optima^®^ from Sigma-Aldrich, polyvinylidene fluoride (PVDF) syringe filters (13 mm diameter, 0.2 µm pore) from Whatman (GE Healthcare, Buckinghamshire, UK), glass Pasteur pipettes, and 2 mL vials with screw caps and septa from Agilent Technologies (Santa Clara, CA, USA), were also used for the extraction method.

The determination of rutin, quercetin and apigenin was performed in a HPLC consisting of a Varian ProStar 230 solvent delivery and a Varian ProStar 330 photodiode array detector (PDA) (Palo Alto, CA, USA). The chromatographic system was equipped with a manual injection system, including a Rheodyne 7725i valve with a 5 μL loop, both supplied by Supelco (Bellefonte, PA, USA). For the chromatographic separation, a Purosphere STAR RP-18e (150 mm × 4.6 mm × 5 μm) column provided by Merck (Darmstadt, Germany), and protected by a Pelliguard LC-18 guard column (Supelco), were used. The injection in the HPLC system was manually carried out using a 100 µL Hamilton syringe (Reno, NV, USA).

Excel (Microsoft Office, v.2016) and STATGRAPHICS^®^ Centurion XV (Statgraphics Technologies, Inc., The Plains, VA, USA) were used for the calibration analysis and statistical calculations.

### 2.3. Procedures

#### 2.3.1. Synthesis of IL-Based Surfactants

##### Synthesis of Monocationic Imidazolium-Type IL-Based Surfactants

Monocationic imidazolium-type IL-based surfactants were synthesized following a previously reported procedure [22]. Briefly, 0.10 mol of either 1-butylimidazole or 1-methylimidazole, 0.11 mol of the corresponding 1-bromoalkane and 20 mL of isopropanol were mixed and refluxed (70 °C, 24 h, stirring). Isopropanol was subsequently removed under vacuum (at 60 °C and 150 mbar) and the product was then dissolved in 25 mL of Milli-Q water. The excess of starting material was extracted 5 times with 15 mL of ethyl acetate. After purification, water was evaporated under vacuum at 80 °C. The product was then further dried in a vacuum oven for 2 days. Figures S1 and S2 of the SM include the ^1^H-NMR spectra and signal assignments for 1-hexadecyl-3-methyl imidazolium bromide ([C_16_MIm^+^][Br^–^]) and 1-hexadecyl-3-butyl imidazolium bromide ([C_16_C_4_Im^+^][Br^–^], and match with those reported in Reference [22].

##### Synthesis of Tricationic Imidazolium IL-Based Surfactant

The tricationic IL-based surfactant 3,3′,3″-octyl-1,1′,1″-(1,3,5)tris(methylene) benzene imidazolium bromide ([(C_8_Im)_3_Bn^3+^]3[Br^–^]) was synthesized following a previously reported procedure [23]. Briefly, 16.68 mmol of imidazole and 62.3 mmol of potassium hydroxide were dissolved in 40 mL of dimethylsulfoxide. Then, 4.45 mmol of 1,3,5-tris(bromomethyl)benzene was added and stirred for 24 h at room temperature. 40 mL of water was added to the reaction mixture and transferred to a separatory funnel, then extracted four times with 40 mL of chloroform. The organic phases were collected, washed several times with water, dried over anhydrous sodium sulfate and filtered. The solvent was partially evaporated under vacuum followed by the addition of excess diethyl ether. Upon addition of excess diethyl ether, a white powder was precipitated, filtered and washed with diethyl ether. The resulting compound was recrystallized using a dichloromethane-diethyl ether solvent mixture and dried at 60 °C for 12 h. 3.14 mmol of the resulting compound and 4.09 mmol of 1-bromooctane in acetonitrile were heated under reflux for 7 days, then the solvent was partially evaporated under vacuum. The solid obtained was filtered and washed several times with diethyl ether. The product was recrystallized using a dichloromethane-diethyl ether solvent mixture followed by removal of the solvent under reduced pressure, and finally dried at 70 °C for 24 h. The ^1^H-NMR spectrum for this IL-based surfactant is shown in Figure S3 of the SM and matches with that reported in Reference [23].

##### Synthesis of Guanidinium-Type IL-Based Surfactants

Guanidinium-type IL-based surfactants were synthesized following a previously reported procedure [24]. Briefly, 19.2 mmol of octylamine and 19.3 mmol of 1H-pyrazole-1-carboxamidine hydrochloride, and 5 mL of ethanol, were refluxed at 35 °C for 48 h under constant stirring. Then, ethanol was removed under vacuum (50 °C, 150 mbar) and the product was washed three times with 5 mL of ethanol. Figures S4 and S5 of the SM include the ^1^H-NMR spectra for octyl guanidinium chloride ([C_8_Gu^+^][Cl^–^]) and decyl guanidinium chloride ([C_10_Gu^+^][Cl^–^]), and match with those reported in Reference [24].

#### 2.3.2. HPLC-PDA Method

The chromatographic separation of the analytes was carried out using acetonitrile, and ultrapure water containing 0.1% (*v/v*) of acetic acid, as mobile phases, and at a constant flow of 1 mL∙min^−1^. The elution gradient started at 10% of acetonitrile (*v/v*) and it was increased to 30% (*v/v*) in 40 min. Then, the percentage of acetonitrile was increased to 100% (*v/v*) in 10 min, and finally, these conditions were held for 2 min. The quantification wavelength for all the analytes was set at 340 nm. The identification of the analytes in the samples was first determined by spiking and injecting extracts of the IL-MA-SLE method under preliminary conditions using the *Passiflora* sp. PS032 sample. During the quantification study, the identification was carried out taking into account the retention times and each UV spectrum, which were compared with those obtained using standard solutions of the analytes under the same HPLC-PDA conditions.

#### 2.3.3. IL-MA-SLE Method Using Ionic Liquid-Based Surfactants

In the MA-SLE method, a specific amount of lyophilized leaves was placed into a 25 mL centrifuge tube, and a specific volume of an aqueous solution of an IL-based surfactant at a certain concentration was added. Then, the analytes were extracted by microwave-assisted extraction by setting the microwave (MW) irradiation power at 50 W, at an adequate temperature, and for a specific time. Finally, the extract was filtered through a PVDF syringe filter and collected in a 2 mL vial. Three repetitions of each extraction were performed. Figure S6A of the SM includes a general scheme of the extraction procedure. Under optimum conditions, 50 mg of leaves and 525 µL of an aqueous solution of the IL-based surfactant at a concentration 50 times higher than the respective CMC were mixed. The extraction was accomplished in the microwaves at 30 °C, 50 W and for 10.5 min.

#### 2.3.4. UA-SLE Method Using Methanol

The conventional extraction method (for the comparison study) was performed according to the previous work [20]. 100 mg of lyophilized leaves were mixed with 10 mL of methanol/water 70% (*v/v*) in a centrifuge tube. The tube was closed and placed in an ultrasonic bath for 15 min at room temperatutre. At the end of the process, the supernatant was collected in another tube and centrifuged at 2515× *g* for 10 min. Thereafter, the supernatant was poured in a separatory funnel and 5 mL of hexane was added in order to perform liquid-liquid extraction of chlorophylls by gently shaking for 5 min. The hydroalcoholic extract was collected into a round bottom flask and evaporated under vacuum at 50 °C until a final volume of 1 mL was obtained. Finally, 1 mL of methanol was added, and it was filtered through a PVDF syringe filter and collected in a vial for its analysis. The extractions were performed in triplicate to obtain the mean concentration values. Figure S6B of the SM includes a scheme of this procedure.

#### 2.3.5. IL-MA-SLE Optimization using Experimental Designs

Response surface methodology (RSM) using that Box-Behnken experimental design (BBD) was employed to find the optimal conditions for the extraction procedure, while intending an improvement of the entire extraction yield. Four factors at three levels were studied, including MW irradiation time (X_1_), extraction temperature (X_2_), liquid-solid (l/s, aqueous IL solution and plant material) ratio (X_3_) and IL-based surfactant concentration in the aqueous solution (X_4_). The peak area obtained for each analyte was used as a response variable (Y).

The number of experiments is calculated as *n* = 2k (k − 1) + C_0_, where k is the number of factors and C_0_ is the number of center points. The operating values were calculated using the following equation:

(1) $$C_{i}=\frac{X_{i}-X_{i}^{0}}{\Delta X_{i}}\;\alpha$$

where *C_i_* is the coded value for the level of factor *i*, *X_i_* is its real value in an experiment, $X_{i}^{0}$ is the real value at the center of the experimental domain, Δ*X_i_* is the step of variation of the real value and *α* is the coded value limit for each factor.

The response surfaces obtained fit the following second order multivariate regression equation, which correlates the variables and the extraction yield:

(2) $$Y=\beta_{0}+\overset{k}{\underset{i=1}{\sum }}\beta_{i}x_{i}+\overset{k}{\underset{i=1}{\sum }}\beta_{ii}x_{i}^{2}+\overset{k-1}{\underset{i=1}{\sum }}\overset{k}{\underset{j>i}{\sum }}\beta_{ij}x_{i}x_{j}$$

where *x_i_* and *x_j_* represent independent variables, *Y* is the response variable and *β*_0_, *β_i_*, *β_ii_* and *β_ij_* are constants, regression coefficients of one term, quadratic terms and interaction terms, respectively. Considering that four variables are studied, the previous equation takes the following form:

(3) $$Y=\beta_{0}+\beta_{1}x_{1}+\beta_{2}x_{2}+\beta_{3}x_{3}+\beta_{4}x_{4}+\beta_{12}x_{1}x_{2}+\beta_{13}x_{1}x_{3}+\beta_{14}x_{1}x_{4}+\beta_{23}x_{2}x_{3}+\beta_{24}x_{2}x_{4}+\beta_{34}x_{3}x_{4}+\;\beta_{11}x_{1}^{2}+\beta_{22}x_{2}^{2}+\beta_{33}x_{3}^{2}+\beta_{44}x_{4}^{2}$$

For the optimization of the method, the [C_16_C_4_Im^+^][Br^–^] IL was selected, and the optimum conditions were extrapolated to the other tested ILs. STATGRAPHICS^®^ Centurion XV software was used to obtain the response surfaces and optimum conditions.

## 3. Results and Discussion

### 3.1. Optimization of IL-MA-SLE Method by RSM

Considering the promising results obtained in our previous study related to the extraction of phenolic compounds from *Vitis vinifera* leaves by an IL-MA-SLE method using [C_16_C_4_Im^+^][Br^–^][20], this IL was selected to optimize the extraction procedure in the current study. Ultimately, the purpose was to use this IL as a screening solvent but intending the further use of ILs with lower toxicity and higher analytical performance. Three flavonoids (apigenin, rutin, quercetin) were selected as target analytes given their significant presence in passion fruit and mango leaves, as it has been previously reported in the literature [15,16,17,18,25]. Table S2 of the SM includes the main physicochemical characteristics of these analytes, together with their structures. The method optimization was performed using an experimental design to reduce the number of experiments, save reagents and plant materials, and to determine the interactions among variables that affect the extraction efficiency towards the target analytes. Amongst all the leaves samples, the *Passiflora* sp. PS032 sample was used as a representative matrix to carry out the optimization since it is the most successful and consumed line of this plant in the Canary Island.

Several parameters can be considered to select the best experimental conditions in an IL-MA-SLE method, such as MW irradiation conditions, amount of sample, amount of extraction solution and IL-based surfactant concentration. According to preliminary tests and other methods reported in the literature dealing with MA-SLE methods [6,26,27,28,29], four main factors were selected for optimization: MW irradiation time, MW irradiation temperature, liquid to solid ratio (l/s) and concentration of IL-based surfactant. MW irradiation power was fixed at 50 W in order to save energy. Besides, high MW power produces a high temperature inside the plant material, which may destroy some of the target compounds, thus reducing the extraction efficiency [30]. The sample amount was fixed to 50 mg, to meet green requirements, while the IL-based surfactant aqueous solution volume was varied to evaluate the effect of the l/s ratio.

The Box-Behnken statistical design was used to optimize the method. This design for four variables (studied at three levels each), and with three repetitions of the center point, comprises 27 experiments arranged in orthogonal blocks [31]. In comparison with other response surface designs (central composite (CCD), Doehlert matrix (DM) and three-level full factorial design), BBD is the most efficient, together with DM. Another advantage of BBD is that it does not contain combinations of factors and levels and, therefore, all factors are simultaneously evaluated at their highest and lowest levels. Furthermore, in comparison with CCD, the number of required experiments for all factors is much lower [31]. The coded and operating values of the BBD of the present study are listed in Table S3 of the SM. The limit values of the factors were selected according to previous studies [6,26,27,28,29,30] and a few preliminary tests, while the dependent variable used to evaluate the extraction efficiency in the experimental design was the peak area of the three target analytes. Thus, the ranges assessed for the different variables were 5–30 min for extraction time, 30–80 °C for extraction temperature, 10–50 mL∙g^−1^ for l/s ratio and 0.9–45 mM (from the CMC to 50 times the CMC) for the [C_16_C_4_Im^+^][Br^–^] IL-based surfactant concentration.

**Figure 1:**
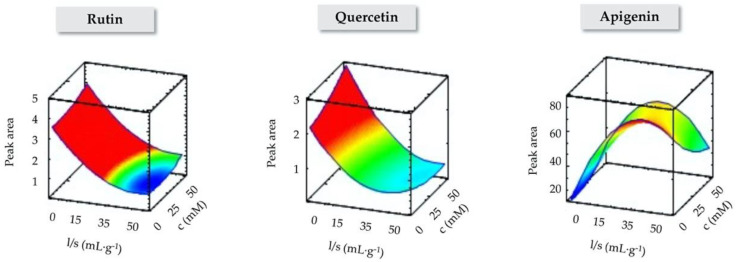
Representative response surfaces described by the second order multivariate regression equation, obtained for each target flavonoid, showing the dependency of the peak area with the IL concentration (c, mM) and the liquid/solid (l/s, mL·g^−1^) ratio.

The obtained constant and coefficients for the second order multivariate regression equation that describes the estimated response surfaces are listed in Table S4 of the SM for each target flavonoid. The three-dimensional plots presenting the dependency of the peak area with the studied variables were constructed and are shown in Figure S7 of the SM, while those corresponding to the effect of l/s ratio and IL-based surfactant concentration are also included in Figure 1 as representative examples. The effects of chosen parameters on the extraction efficiency of target compounds, and interactions between them, can be estimated from the shape of the three-dimensional response surface. For example, as it can be observed in the representative response surfaces (Figure 1), the peak area of rutin and quercetin decreased with the increase of the l/s ratio. However, the behavior is totally different for apigenin since the extraction efficiency increases as the l/s ratio increases, and then it starts to decrease.

With the aim of finding trends, and particularly, trying to understand which of the studied factors significantly affect the IL-MA-SLE extraction efficiency, an analysis of variance (ANOVA) was performed, with results shown in Table S5 of the SM. The determination coefficients (R^2^) of the regressions indicate that the fitted models explain 89% of the variability in rutin, 58% in quercetin and 37% in apigenin. Thus, the model is well fitted for rutin, but less for quercetin and apigenin. According to the *p*-values, the major statistically significant factor influencing the peak area was the l/s ratio in the linear (*β*_3_) and quadratic terms (*β*_13_, *β*_23_, *β*_33_ and *β*_34_) for all the analytes, except for apigenin, as previously observed from Figure 1. According to the *p*-values, the extraction time is an important factor influencing the extraction yield of apigenin (*β*_1_ and *β*_11_). For all the analytes, the extraction yield firstly increases with increasing extraction times. However, with further increase of time, the extraction yield decreases, which is particularly significant for apigenin, maybe due to the decompositions of analytes.

The optimum conditions for each analyte predicted by the statistical software are included in Table S6 of the SM. In general, rutin and quercetin present similar optimum conditions: low extraction times, temperature and l/s ratio, and the maximum IL-based surfactant concentration (50 times the CMC). In the case of apigenin, best results were obtained with higher extraction times and l/s ratios, the highest extraction temperature and the lowest IL-based surfactant concentration (CMC value). In order to benefit the highest number of analytes, a compromise solution was proposed and the following optimum conditions were chosen for further extractions: 10 min of extraction time, 30.0 °C for the extraction temperature, 10.5 mL∙g^−1^ of l/s ratio and 50 times the CMC for the IL-based concentration. The l/s ratio was kept at this value to favor rutin and quercetin, for which this variable was especially significant. The extraction time, which affected apigenin the most, was set at an intermediate value amongst the optimum times, which were very similar. Due to the non-significant influence of temperature, it was set at the minimum value to save energy, while the highest IL-based surfactant concentration was used due to its positive effect at low temperatures for rutin and quercetin.

### 3.2. Analytical Performance of the IL-MA-SLE-HPLC-PDA Method

Once the IL-MA-SLE method was optimized, and the target compounds were correctly identified in the chromatograms, the quantification of the analytes was required in order to determine their concentration in the different samples. The external calibration method was used for the quantification of the analytes in the samples. Linear range, calibration sensitivity (determined as the calibration slope), inter-day precision, determination coefficients (R^2^), limits of detection (LODs) and limits of quantification (LOQs) of the chromatographic method were determined. The results of calibration data together with several analytical quality parameters are shown in Table S7 of the SM. The method exhibits good linearity with R^2^ values higher than 0.9988, within the concentration range of 0.1–500 mg·L^−1^ for rutin, 0.05–500 mg·L^−1^ for quercetin and 0.03–500 mg·L^−1^ for apigenin. LODs were experimentally obtained by decreasing the concentration of the analytes in the standard until a signal-to-noise ratio (S/N) of 3 was obtained, while the LOQs were estimated as 10/3 times the LODs and then experimentally verified. Thus, chromatographic LODs were 40, 20 and 10 µg∙L^−1^ for rutin, quercetin and apigenin, respectively. The chromatographic LOQs ranged from 30 to 100 µg∙L^−1^. In order to assess the precision of the method, standard solutions at 30 mg·L^−1^ were analyzed by HPLC-PDA three consecutive times in the same day, and in three non-consecutive days. The proposed method presents good intermediate precision, with RSD values lower than 3.22%.

### 3.3. Evaluation of Different IL-Based Surfactants in the IL-MA-SLE-HPLC-PDA Method

The effect of the IL structure in the extraction of the target flavonoids from plant leaves was evaluated using six IL-based surfactants. Table 1 shows the chemical structure and some of the most important properties of the IL-based surfactants evaluated in this study, including their CMC values [21,22,23,32,33,34]. All of them were compatible with HPLC analysis due to their solubility in the mobile phase, thus not requiring a back-extraction of the IL extract before the injection in the instrument. They were selected considering different factors. Imidazolium ILs have been widely used for the extraction of different analytes from different samples [5,7,26,30,35,36]. The effect of the secondary alkyl chain was assessed by using [C_16_MIm^+^][Br^–^] and [C_16_C_4_Im^+^][Br^–^]. In the case of multicationic IL-based surfactants, despite their interesting characteristics, they have been scarcely evaluated in extraction procedures, thus [(C_8_Im)_3_Bn^3+^]3[Br^–^] was selected. Considering the recent concern on the toxicity of most common imidazolium ILs [37], other cationic cores were evaluated, including pyridinium [C_16_Py^+^][Br^–^] and alkyl guanidinium chloride IL-based surfactants. In the case of guanidinium ILs, which have been recently reported as low cytotoxic ILs [21,34], different alkyl chains were also studied ([C_8_Gu^+^][Cl^–^] and [C_10_Gu^+^][Cl^–^]). It is also important to highlight the environmentally friendliness in the synthetic procedure for the guanidinium ILs in comparison with that of imidazolium ILs, the latter requiring the use of toxic organic solvents (e.g., chloroform).

Given the different structures of the tested ILs, they present different CMC values, which range from 0.61 mM for [C_16_MIm^+^][Br^–^] to 44.6 mM for [C_8_Gu^+^][Cl^–^]. Therefore, imidazolium IL-based surfactants present the lowest CMC values and lower amounts of IL are required to take advantage of their surface-active properties in comparison with guanidinium ILs. However, the ILs with guanidinium moieties present safer toxicological profiles. Apart from these properties, several studies have pointed out that the structure of ILs has a significant effect on the extraction efficiency of target analytes [27,30,38,39]. In general, the anion as well as the length of IL alkyl chain affects water miscibility, thereby it affects the extraction efficiency of target compounds [30,40,41,42]. Thus, ILs with halide anions, such as [Cl^–^] or [Br^–^], are miscible with water in any proportion, but those ILs containing [PF_6_^–^] are mostly hydrophobic. On the other hand, increasing the alkyl chain length of the ILs also increases the hydrophobicity and viscosity of the ILs, whereas densities and surface tension values decrease [43].

A screening study was carried out to evaluate the extraction performance of the selected IL-based surfactants in the optimized IL-MA-SLE method and using PS032 and Sweet tart samples as representative leaves matrices for passion fruit and mango, respectively. The optimum conditions previously obtained were used for the remaining IL-based surfactants. Therefore, the extraction time, temperature and l/s ratio were the same, while the IL-based surfactant concentration was different for each IL since it depends on their CMC value. Thus, 30.5 mM was used for [C_16_MIm^+^][Br^–^], 45 mM for [C_16_C_4_Im^+^][Br^–^], 36 mM for [C_16_Py^+^][Br^–^], 2230 mM for [C_8_Gu^+^][Cl^–^], 930 mM for [C_10_Gu^+^][Cl^–^] and 115 mM for [(C_8_Im)_3_Bn^3+^]3[Br^–^], in all cases being 50 times higher than their respective CMC values.

**Figure 2:**
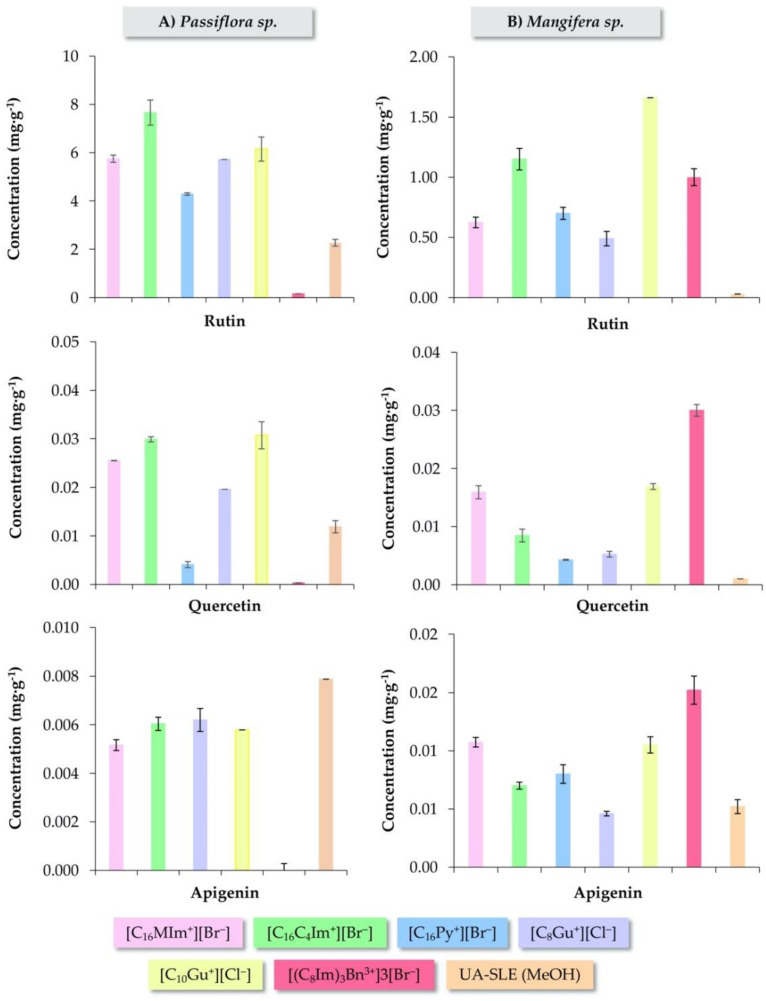
Extraction performance of the IL-MA-SLE-HPLC-PDA method when using different IL-based surfactants for the determination of rutin, quercetin and apigenin, in *Passiflora* sp. (PS032) and *Mangifera* sp. (Sweet tart) leaves, respectively. Extractions were performed in triplicate under the optimum experimental conditions described in Section 2.3.3.

The obtained results are included in Figure 2, showing the concentration obtained for each flavonoid when using the different IL-based surfactants as extractants. It is important to highlight the good precision in the determination of the analytes when using all the IL-based surfactants, with RSD values lower than 2.48%. As it can be observed, within the same cationic core, the longer the alkyl chain length, the better extraction efficiency for all the analytes. Thus, [C_10_Gu^+^][Cl^–^] provided better results than its analogue with a chain of 8 carbon atoms for both samples, while the imidazolium IL-based surfactant with the longest substituents exhibited better extraction performance for all the analytes in the *Passiflorora* sp. sample, and for rutin in the *Magnifera* sp. sample. In the case of the tricationic IL-based surfactant, it provided the lowest extraction efficiency when analyzing the *Passiflorora* sp. leaves and, indeed, it was not able to extract apigenin. However, this multicationic IL-based surfactant presented the best results for the extraction of apigenin and quercetin in the *Magnifera* sp. samples, particularly for quercetin. In the case of the pyridinium IL, the extraction efficiency was slightly lower in comparison with the remaining IL-based surfactants, except for apigenin in both samples, for which they presented similar results. Therefore, in general, it is clear that [C_16_C_4_Im^+^][Br^–^] and [C_10_Gu^+^][Cl^–^] are the most efficient extractants for passion fruit leaves, while the tricationic IL and [C_10_Gu^+^][Cl^–^] were the best for mango leaves, in comparison with the remaining IL-based surfactants evaluated. Given the significantly different behavior observed for both samples (regardless of their different flavonoids content), it is difficult to determine that a single or a specific type of IL-based surfactant will provide the best results for this application. When comparing plant extracts, it must be taken into account that they have a quite complex composition. Therefore, the origin of the leaves must be considered when evaluating different IL-based surfactant characteristics to enhance the extraction of flavonoids from any type of plant material.

Considering these results and with the aim of and favoring the best extraction performance for both type of samples and improving the sustainability of the method, [C_10_Gu^+^][Cl^–^] was selected as the optimum extraction IL-based surfactant in further research. This IL presents low cytotoxicity in comparison with the imidazolium ILs, as it has been previously reported [21]. Despite that higher amounts of IL are required for [C_10_Gu^+^][Cl^–^] due to its higher CMC value, the amount of this IL-based surfactant for each extraction is only 488 µL, which is still really low. Moreover, [C_16_C_4_Im^+^][Br^–^] and [(C_8_Im)_3_Bn^3+^]3[Br^–^] are solids at room temperature, while [C_10_Gu^+^][Cl^–^] is a liquid, which facilitates its manipulation and the preparation of aqueous solutions.

With the purpose of evaluating the performance of [C_10_Gu^+^][Cl^–^] in the proposed method, a comparison with a more conventional extraction method previously reported [44] was also carried out. The same samples with the same conditions of l/s ratio as the proposed method were extracted three times by a UA-SLE method using methanol. The extracts were further analyzed by HPLC-PDA to obtain the average concentrations of the target flavonoids. The obtained results are also included in Figure 2 for both samples. It can be observed that both methods provided similar relative composition (percentage of each flavonoid concentration with respect to the total content of flavonoids) of rutin, quercetin and apigenin, but showing different concentrations.

Figure S8 of the SM includes the chromatograms of the *Passiflora* sp. PS032 extracts obtained by the IL-MA-SLE-HPLC-PDA method using the [C_10_Gu^+^][Cl^–^], in comparison with the UA-SLE-HPLC-PDA method, with clear differences in the flavonoid signals depending on the method. The conventional UA-SLE method yielded much lower concentrations of the analytes, except for apigenin in *Passiflorora* sp., for which the results were slightly higher. Indeed, as a general statement, the proposed method with IL-based surfactants exhibited higher or comparable yields of rutin, quercetin and apigenin if compared with studies reported in the literature using other extraction methods for the isolation of flavonoids from plant materials [19,25].

Apart from the differences in the extraction performance of both methods, it is important to highlight other advantages of the proposed IL-MA-SLE method over the UA-SLE used for the same application. The proposed method has fewer steps, as shown in Figure S6 of the SM, making the process less tedious and faster due to the elimination of the clean-up step. Our previous study demonstrated that the use of IL-based surfactant aqueous solutions as an extraction solvent avoids the co-extraction of green chlorophylls that could compromise the analytical performance of the chromatographic column [20]. In the current study, the entire IL-MA-SLE consumes less time, 15 min compared to the 45 min required in the UA-SLE method. Moreover, MW power is energetic enough to deal with the plant matrix while allowing a faster diffusion of the target compounds to the solvent [45,46]. Another important advantage of the method proposed in this study in comparison with the more conventional method and our previous study [20] is the use of an IL of low cytotoxicity (which only requires ethanol in the synthetic procedure), thus gaining in greenness over the organic solvent required in the UA-SLE method, and the imidazolium IL utilized in our previous MA-SLE method [20].

Flavonoids present in plant by-products of fruit trees, particularly in leaves, have also been determined through a number of extraction methods using different extraction solvents, including imidazolium-based ILs [27,28,29,30,37,47] in combination with HPLC and UV [27,28,29,30,36] or mass spectrometry (MS) [13,15] detection. Conventional extractions using solvents with different polarity such as ethanol [13,15,25], methanol [19,47] and chloroform [19], are one of the most used extraction techniques for isolation of flavonoids from plant by-products. Conventional Soxhlet extraction is another option to extract flavonoids from plant by-products but it is far more time and energy consuming [11,29]. In comparison with these strategies, our proposed method uses low toxicity IL and requires relatively low or similar volumes of ILs solutions as extraction solvent (~500–2500 µL for 50 mg of plant material) [27,28,29,30]. Therefore, the method proposed in the current study is much more efficient for extraction of flavonoids from plant by-products and it is characterized by its greenness in comparison with other methods, mainly in terms of toxicity of the extraction medium as well as time and energy consumption.

### 3.4. Analysis of Plant Samples under Optimum IL-MA-SLE-HPLC-PDA Conditions

The optimized IL-MA-SLE-HPLC-PDA method using the [C_10_Gu^+^][Cl^–^] IL was applied to the determination of the three target flavonoids in plant leaves samples from *Passiflora* sp. and *Mangifera* sp. (Table S1 of the SM). Initially, seven analytes (rutin, quercetin, apigenin, myricetin, kaempferol, naringin and ellagic acid) were considered, but only the three flavonoids previously studied were detected in all samples. The results are included in Table 2. Rutin, quercetin and apigenin were detected in all passion fruit and mango leaves samples. The concentration of rutin, quercetin and apigenin varied in all the lines of passion fruit leaves from 2.35 to 6.15 mg∙g^−1^, from 0.021 to 0.090 mg∙g^−1^ and from 0.006 to 0.017 mg∙g^−1^. Also, the concentration of rutin, quercetin and apigenin varied in all four cultivars of mango leaves from 0.082 to 0.239 mg∙g^−1^, from 0.006 to 0.044 mg∙g^−1^ and from 0.007 to 0.015 mg∙g^−1^, respectively. It is clear that concentrations of flavonoids among types of plant species are different. The phenolic content of the plant extract is influenced by several factors such as environmental (e.g., temperature, rainfall, day length [48]), harvesting (e.g., season, geographical location growth stage, daily harvest period [49,50,51]) and post-processing of plant material (e.g., drying [52,53]).

The richest line of *Passiflora* sp. leaves in flavonoids was PS032, while the richest cultivar of *Mangifera* sp. leaves in flavonoids was Mun. Extracts from passion fruit leaves were much richer in rutin than extracts from mango leaves (30 times higher), while content of quercetin and apigenin was very similar in both plant leaves. Furthermore, the extracts from passion fruit 18PS003 had the lowest concentration of flavonoids among all lines of passion fruit leaves. The extracts of Mango cultivars Gomera 1 and 3 were very similar due to their same origin (Gomera island). Mango Sweet Tart had lower content of flavonoids compared to Mun but higher compared to Gomera 1 and 3. Considering the total concentration of flavonoids determined, *Passiflora* sp. leaves exhibit a great potential to be exploited for its valorization, particularly due to its high content of rutin. Rutin was previously determined in 3 species of passion fruit leaves from Brazil in concentrations from 0.57 to 3.48 mg∙g^−1^ [25], which is similar or slightly lower in comparison with those analyzed in the present study. Apart from quercetin and apigenin, it has been previously reported that *Passiflora* sp. by-products are also rich in orientin, isoorientin, vitexin and isovitexin [16,25]. El-Hawary et al. determined the flavonoid content of 8 species of mango leaves from Egypt [54]. They determined 9 flavonoids in extracts, including rutin, quercetin and apigenin, which were quantified at concentrations from 0.67 to 6.99 mg∙g^−1^, from 0.04 to 0.14 mg∙g^−1^ and from 0.005 to 0.17 mg∙g^−1^, respectively. In that particular case, the content of all analytes was higher than in the samples from Canary Islands analyzed in this study.

## 4. Conclusions

Six IL-based surfactants containing different cation moieties (imidazolium-, guanidinium- and pyridinium-type ILs), alkyl chains, and even number of cationic moieties, were successfully used in a MA-SLE method in combination with HPLC-PDA to evaluate the influence of the structure and composition of the IL on the extraction performance towards flavonoids. The result showed that the structure of ILs has a significant effect on the extraction efficiency of target analytes, while the origin of the plant material (leaves in this particular case) was also an important factor to consider when evaluating their performance. It was observed that for ILs within the same cationic core, the longer the alkyl chain length, the better extraction efficiency for all the analytes. [C_16_C_4_Im^+^][Br^–^] and [C_10_Gu^+^][Cl^–^] were the most efficient extractants in comparison with the other IL-based surfactants evaluated for *Passiflora* sp. leaves, while [C_16_C_4_Im^+^][Br^–^] and [(C_8_Im)_3_Bn^3+^]3[Br^–^] were the most efficient extractants for *Mangifera* sp. leaves. All three IL-based surfactants provided similar results, but with the aim of improving the sustainability of the method, [C_10_Gu^+^][Cl^–^] was selected as the optimum extractant due to its low cytotoxicity.

Apart from being simpler and faster, the greenness of the IL-MA-SLE method is given by the use of MW energy and the low cytotoxicity of IL-based surfactant, together with the high extraction efficiencies achieved in the entire method. This group of advantages highlights the proposed methodology over the conventional UA-SLE method that uses methanol as an extraction medium.

Regarding contents in the leaves analyzed, it is important to mention that 3 flavonoids were determined in 5 different lines of *Passiflora* sp. leaves and in 4 different cultivars of *Mangifera* sp. leaves using the proposed method. Concentrations of flavonoids in the by-products of both species were different, with *Passiflora* sp. having the highest flavonoids content. Moreover, different flavonoid content in the leaves extracts was obtained among all cultivars or lines for each plant, which was not that significant but still observable.

## Figures and Tables

**Table 1:** Several characteristics of IL-based surfactants evaluated in the IL-MA-SLE-HPLC-PDA method for the extraction of flavonoids from plants.

IL Full Name [IL Abbreviation]	Structure	State	Molecular Weight (g∙mol^−1^)	CMC ^a^ (mM)/Ref.
*1-hexadecyl−3-methyl imidazolium bromide*				
[C_16_MIm^+^][Br^–^]	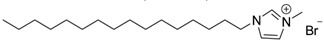	Solid	386.9	0.61/[32]
*1-hexadecyl-3-butyl imidazolium bromide*				
[C_16_C_4_Im^+^][Br^–^]	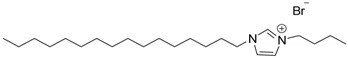	Solid	428.3	0.10/[22]
*Hexadecyl pyridinium bromide*				
[C_16_Py^+^][Br^–^]	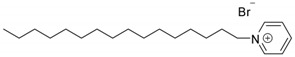	Solid	384.4	0.72/[33]
*Octyl guanidinium chloride*				
[C_8_Gu^+^][Cl^–^]	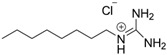	Liquid	206.5	44.6/[34]
*Decyl guanidinium chloride*				
[C_10_Gu^+^][Cl^–^]	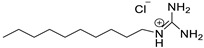	Liquid	234.5	18.6/[21]
*3,3′,3″-octyl-1,1′,1″-(1,3,5)tris(methylene) benzene imidazolium bromide*				
[(C_8_Im)_3_Bn^3+^]3[Br^-^]	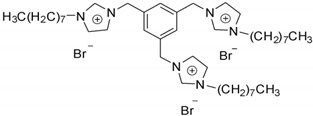	Solid	849.8	2.30/[23]

^a^ Critical micelle concentration value reported in the literature.

**Table 2:** Flavonoids content (in mg·g^−1^) of passion fruit and mango leaves from Canary Islands, analyzed by the proposed IL-MA-SLE-HPLC-PDA method, using an aqueous solution of the [C_10_Gu^+^][Cl^–^] IL-based surfactant as extractant.

Plant	Rutin (RSD *)	Quercetin (RSD *)	Apigenin (RSD *)
*Passiflora* sp.			
PS032	6.15 (8.0%)	0.031 (9.0%)	0.006 (5.0%)
17PS009	4.15 (8.0%)	0.046 (7.0%)	0.006 (0.5%)
PS003	4.51 (5.0%)	0.021 (9.0%)	0.010 (8.0%)
17PS008	2.59 (7.0%)	0.090 (2.0%)	0.017 (2.0%)
18PS003	2.35 (3.0%)	0.036 (6.0%)	0.008 (6.5%)
*Mangifera* sp.			
Sweet Tart	0.163 (2.0%)	0.031 (1.5%)	0.015 (8.5%)
Mun	0.239 (0.3%)	0.044 (3.0%)	0.011 (9.0%)
Gomera 1	0.082 (2.0%)	0.006 (1.7%)	0.007 (1.2%)
Gomera 3	0.082 (1.2%)	0.011 (2.0%)	0.008 (2.0%)

* relative standard deviation (*n* = 3).

## Supplementary Materials

The following are available online. **Figure S1:** ^1^H-NMR spectrum (DMSO-*d_6_*, 600 MHz) of [C_16_MIm^+^][Br^–^]. δ (ppm): 0.86 (t, *J* = 6.88 Hz, 3 H), 1.24 (m, 26 H), 1.77 (m, 2 H), 3.85 (s, 3 H), 4.15 (t, 2 H), 7.69 (s, 1 H), 7.76 (s, 1 H), 9.11 (s, 1 H). **Figure S2:** ^1^H-NMR spectrum (DMSO-*d_6_*, 600 MHz) of [C_16_C_4_Im^+^][Br^–^]. δ (ppm): 0.77–0.98 (dt, 6 H), 1.14–1.35 (m, 28 H), 1.69–1.87 (m, 4 H), 4.17 (dt, 4 H), 7.80 (s, 2 H), 9.24 (s, 1 H). **Figure S3:** ^1^H-NMR spectrum (DMSO-*d_6_*, 600 MHz) of [(C_8_Im)_3_Bn^3+^]3[Br^–^]. δ (ppm): 0.86 (t, *J* = 6.88 Hz, 9 H), 1.26 (m, 30 H), 1.69–1.89 (m, 6 H), 4.20 (t, *J* = 7.34 Hz, 6 H), 5.47 (s, 6 H), 7.57 (s, 3 H), 7.84 (m, 6 H), 9.50 (s, 3 H). **Figure S4:** ^1^H-NMR spectrum (DMSO-*d_6_*, 500 MHz) of [C_8_Gu^+^][Cl^–^]. δ (ppm): 0.77–0.90 (t, 3 H), 1.16–1.33 (sa, 10 H), 1.36–1.52 (m, 2 H), 3.08 (t, *J* = 7.03 Hz, 2 H), 6.23 (t, *J* = 1.96 Hz, 1 H), 7.58 (s, 2 H). **Figure S5:** ^1^H-NMR spectrum (DMSO-*d_6_*, 500 MHz) of [C_10_Gu^+^][Cl^–^]. δ (ppm): 0.85 (t, *J* = 6.76 Hz, 3 H), 1.24 (sa, 12 H), 1.37–1.50 (m, 2 H), 3.08 (t, *J* = 6.99 Hz, 2 H), 6.20–6.29 (m, 1 H), 7.59 (s, 2 H). **Figure S6:** Scheme of (A) the proposed IL-MA-SLE-HPLC-PDA method, when performed under optimum conditions, and (B) the conventional UA-SLE-HPLC-PDA method, both used for the extraction of flavonoids from plant leaves. **Figure S7:** Obtained response surfaces as described by the second order multivariate regression equation for each target flavonoid, presenting the dependency of the peak area with the studied variables. **Figure S8:** Representative chromatograms obtained for the analysis of *Passiflora* sp. PS032 leaves using the IL-MA-SLE-HPLC-PDA method with the [C_10_Gu^+^][Cl^−^], in comparison with the chromatogram obtained when using the conventional UA-SLE-HPLC-PDA method. Peak 1: rutin, peak 2: quercetin, peak 3: apigenin. **Table S1:** Plant identification and anatomy of *Passiflora* sp. and *Mangifera* sp. leaves analyzed in this study for the quantification of flavonoids. **Table S2****:** Chemical structures and physicochemical properties of the flavonoids determined in this study, obtained from the SciFinder^®^ 2020 database. **Table S3:** Matrix of experiments of the Box-Behnken design used for the optimization of the IL-MA-SLE method, including the coded and the operating values. **Table S4:** Obtained values for the constant and coefficients of the second order multivariate regression equation for the fitted response surfaces of rutin, quercetin and apigenin, as target flavonoids. **Table S5:** Several parameters obtained from the analysis of variance (ANOVA) of the experimental results using the BBD. **Table S6:** Optimum values obtained with the BBD for each target flavonoid when using the IL-MA-SLE method with the [C_16_C_4_Im^+^][Br^–^] IL-based surfactant, and *Passiflora flavicarpa* (PS032) as a model sample (50 mg). **Table** **S7****:** Several analytical quality parameters of the HPLC-PDA method for the determination of rutin, quercetin and apigenin.

## References

[1] Armenta S., Garrigues S., De la Guardia M. (2008). Green analytical chemistry. TrAC.

[2] Pacheco-Fernández I., Pino V. (2019). Green solvents in analytical chemistry. Curr. Opin. Green Sustain. Chem..

[3] Ullah H., Wilfred C.D., Shaharun M.S. (2019). Ionic liquid-based extraction and separation trends of bioactive compounds from plant biomass. Sep. Sci. Technol..

[4] Passos H., Freire M.G., Coutinho J.A. (2014). Ionic liquid solutions as extractive solvents for value-added compounds from biomass. Green Chem..

[5] Liu Z., Chen Z., Han F., Kang X., Gu H., Yang L. (2016). Microwave-assisted method for simultaneous hydrolysis and extraction in obtaining ellagic acid, gallic acid and essential oil from Eucalyptus globulus leaves using Brönsted acidic ionic liquid [HO3S (CH2) 4mim] HSO4. Ind. Crop. Prod..

[6] Liu X., Wang Y., Kong J., Nie C., Lin X. (2012). Application of ionic liquids in the microwave-assisted extraction of quercetin from Chinese herbal medicine. Anal. Methods.

[7] Zhao C., Lu Z., Li C., He X., Li Z., Shi K., Yang L., Fu Y., Zu Y. (2014). Optimization of ionic liquid based simultaneous ultrasonic-and microwave-assisted extraction of rutin and quercetin from leaves of velvetleaf (Abutilon theophrasti) by response surface methodology. Sci. World J..

[8] Pacheco-Fernández I., González-Hernández P., Pino V., Ayala J.H., Afonso A.M. (2017). Ionic Liquid-Based Surfactants: A Step forward. Ionic Liquid Devices.

[9] Villacís-Chiriboga J., Elst K., Van Camp J., Vera E., Ruales J. (2020). Valorization of byproducts from tropical fruits: Extraction methodologies, applications, environmental, and economic assessment: A review (Part 1: General overview of the byproducts, traditional biorefinery practices, and possible applications). Compr. Rev. Food Sci. Food Saf..

[10] Altınok E., Palabiyik I., Gunes R., Toker O.S., Konar N., Kurultay S. (2020). Valorisation of grape by-products as a bulking agent in soft candies: Effect of particle size. LWT Food Sci. Technol..

[11] Jangra A., Pawar B. (2019). Quantification of Flavonoids from different Parts of Grapefruit (Citrus x Paradisi) from different Extraction Methods. JASFT.

[12] Pimentel-Moral S., de la Luz Cádiz-Gurrea M., Rodríguez-Pérez C., Segura-Carretero A. (2020). Recent advances in extraction technologies of phytochemicals applied for the revaluation of agri-food by-products. Functional and Preservative Properties of Phytochemicals.

[13] Hanganu D., Olah N.K., Pop C.E., Vlase L., Oniga I., Ciocarlan N., Matei A., Puscas C., Silaghi-Dumitrescu R., Benedec D. (2019). Evaluation of Polyphenolic Profile and Antioxidant Activity for Some Salvisa Species. Farmacia.

[14] Fonseca L.R.D., Rodrigues R.D.A., Ramos A.D.S., da Cruz J.D., Ferreira J.L.P., Silva J.R.D.A., Amaral A.C.F. (2020). Herbal Medicinal Products from Passiflora for Anxiety: An Unexploited Potential. Sci. World J..

[15] Sakalem M.E., Negri G., Tabach R. (2012). Chemical composition of hydroethanolic extracts from five species of the Passiflora genus. Rev. Bras. Farmacogn..

[16] Abourashed E.A., Vanderplank J.R., Khan I.A. (2002). High-speed extraction and HPLC fingerprinting of medicinal plants—I. Application to Passiflora flavonoids. Pharm. Biol..

[17] Umamahesh K., Sivudu S.N., Reddy O.V.S. (2016). Evaluation of antioxidant activity, total phenolics and total flavonoids in peels of five cultivars of mango (Mangifera indica) fruit. J. Med. Plants Stud..

[18] Kanwal Q., Hussain I., Siddiqui H.L., Javaid A. (2009). Flavonoids from mango leaves with antibacterial activity. J. Serb. Chem. Soc..

[19] Seal T. (2016). Quantitative HPLC analysis of phenolic acids, flavonoids and ascorbic acid in four different solvent extracts of two wild edible leaves, Sonchus arvensis and Oenanthe linearis of North-Eastern region in India. J. Appl. Pharm. Sci..

[20] Mastellone G., Pacheco-Fernández I., Rubiolo P., Pino V., Cagliero C. (2020). Sustainable Micro-Scale Extraction of Bioactive Phenolic Compounds from Vitis vinifera Leaves with Ionic Liquid-Based Surfactants. Molecules.

[21] Pacheco-Fernández I., Pino V., Lorenzo-Morales J., Ayala J.H., Afonso A.M. (2018). Salt-induced ionic liquid-based microextraction using a low cytotoxic guanidinium ionic liquid and liquid chromatography with fluorescence detection to determine monohydroxylated polycyclic aromatic hydrocarbons in urine. Anal. Bioanal. Chem..

[22] Baltazar Q.Q., Chandawalla J., Sawyer K., Anderson J.L. (2007). Interfacial and micellar properties of imidazolium-based monocationic and dicationic ionic liquids. Colloids Surf. A.

[23] Nacham O., Martín-Pérez A., Steyer D.J., Trujillo-Rodríguez M.J., Anderson J.L., Pino V., Afonso A.M. (2015). Interfacial and aggregation behavior of dicationic and tricationic ionic liquid-based surfactants in aqueous solution. Colloids Surf. A.

[24] El Hankari S., Hesemann P. (2012). Guanidinium vs. Ammonium Surfactants in Soft-Templating Approaches: Nanostructured Silica and Zwitterionic i-Silica from Complementary Precursor–Surfactant Ion Pairs. Eur. J. Inorg. Chem..

[25] Gomes S.V., Portugal L.A., dos Anjos J.P., de Jesus O.N., de Oliveira E.J., David J.P., David J.M. (2017). Accelerated solvent extraction of phenolic compounds exploiting a Box-Behnken design and quantification of five flavonoids by HPLC-DAD in Passiflora species. Microchem. J..

[26] Li C., Lu Z., Zhao C., Yang L., Fu Y., Shi K., He X., Li Z., Zu Y. (2015). Ionic-liquid-based ultrasound/microwave-assisted extraction of 2, 4-dihydroxy-7-methoxy-1, 4-benzoxazin-3-one and 6-methoxy-benzoxazolin-2-one from maize (Zea mays L.) seedlings. J. Sep. Sci..

[27] Wei Z., Zu Y., Fu Y., Wang W., Luo M., Zhao C., Pan Y. (2013). Ionic liquids-based microwave-assisted extraction of active components from pigeon pea leaves for quantitative analysis. Sep. Pur. Technol..

[28] Xu W., Chu K., Li H., Zhang Y., Zheng H., Chen R., Chen L. (2012). Ionic liquid-based microwave-assisted extraction of flavonoids from Bauhinia championii (Benth.) Benth. Molecules.

[29] Zeng H., Wang Y., Kong J., Nie C., Yuan Y. (2010). Ionic liquid-based microwave-assisted extraction of rutin from Chinese medicinal plants. Talanta.

[30] Li C., Zhang J., Zhao C., Yang L., Zhao W., Jiang H., Ren X., Su W., Li Y., Guan J. (2018). Separation of the main flavonoids and essential oil from seabuckthorn leaves by ultrasonic/microwave-assisted simultaneous distillation extraction. R. Soc. Open Sci..

[31] Ferreira S.C., Bruns R., Ferreira H., Matos G., David J., Brandao G., da Silva E.P., Portugal L., Dos Reis P., Souza A. (2007). Box-Behnken design: An alternative for the optimization of analytical methods. Anal. Chim. Acta.

[32] Vanyur R., Biczok L., Miskolczy Z. (2007). Micelle formation of 1-alkyl-3-methylimidazolium bromide ionic liquids in aqueous solution. Colloids Surf. A.

[33] Asakawa T., Kitano H., Ohta A., Miyagishi S. (2001). Convenient estimation for counterion dissociation of cationic micelles using chloride-sensitive fluorescence probe. J. Colloid Interface Sci..

[34] Pacheco-Fernández I., Pino V., Ayala J.H., Afonso A.M. (2018). Guanidinium ionic liquid-based surfactants as low cytotoxic extractants: Analytical performance in an in-situ dispersive liquid–liquid microextraction method for determining personal care products. J. Chromatogr. A.

[35] Liu Z., Qiao L., Gu H., Yang F., Yang L. (2017). Development of Brönsted acidic ionic liquid based microwave assisted method for simultaneous extraction of pectin and naringin from pomelo peels. Sep. Pur. Technol..

[36] Gu H., Chen F., Zhang Q., Zang J. (2016). Application of ionic liquids in vacuum microwave-assisted extraction followed by macroporous resin isolation of three flavonoids rutin, hyperoside and hesperidin from Sorbus tianschanica leaves. J. Chromatogr. B.

[37] Mena I.F., Diaz E., Palomar J., Rodriguez J.J., Mohedano A.F. (2020). Cation and anion effect on the biodegradability and toxicity of imidazolium- and choline-based ionic liquids. Chemosphere.

[38] Lou Z., Wang H., Zhu S., Chen S., Zhang M., Wang Z. (2012). Ionic liquids based simultaneous ultrasonic and microwave assisted extraction of phenolic compounds from burdock leaves. Anal. Chim. Acta.

[39] Yang L., Wang H., Zu Y.-G., Zhao C., Zhang L., Chen X., Zhang Z. (2011). Ultrasound-assisted extraction of the three terpenoid indole alkaloids vindoline, catharanthine and vinblastine from Catharanthus roseus using ionic liquid aqueous solutions. Chem. Eng. J..

[40] Remsing R.C., Swatloski R.P., Rogers R.D., Moyna G. (2006). Mechanism of cellulose dissolution in the ionic liquid 1-n-butyl-3-methylimidazolium chloride: A 13 C and 35/37 Cl NMR relaxation study on model systems. Chem. Commun..

[41] Liang H., Wang W., Xu J., Zhang Q., Shen Z., Zeng Z., Li Q. (2017). Optimization of ionic liquid-based microwave-assisted extraction technique for curcuminoids from Curcuma longa L.. Food Bioprod. Process..

[42] Zhang Q., Zhao S.H., Chen J., Zhang L.W. (2015). Application of ionic liquid-based microwave-assisted extraction of flavonoids from Scutellaria baicalensis Georgi. J. Chromatogr. B.

[43] Huddleston J.G., Visser A.E., Reichert W.M., Willauer H.D., Broker G.A., Rogers R.D. (2001). Characterization and comparison of hydrophilic and hydrophobic room temperature ionic liquids incorporating the imidazolium cation. Green Chem..

[44] Acquadro S., Appleton S., Marengo A., Bicchi C., Sgorbini B., Mandrone M., Gai F., Peiretti P.G., Cagliero C., Rubiolo P. (2020). Grapevine Green Pruning Residues as a Promising and Sustainable Source of Bioactive Phenolic Compounds. Molecules.

[45] Chan C.-H., Yusoff R., Ngoh G.-C., Kung F.W.-L. (2011). Microwave-assisted extractions of active ingredients from plants. J. Chromatogr. A.

[46] Fan Y., Xu C., Li J., Zhang L., Yang L., Zhou Z., Zhu Y., Zhao D. (2018). Ionic liquid-based microwave-assisted extraction of verbascoside from Rehmannia root. Ind. Crop. Prod..

[47] Zorzetto C., Sánchez-Mateo C.C., Rabanal R.M., Lupidi G., Petrelli D., Vitali L.A., Bramucci M., Quassinti L., Caprioli G., Papa F. (2015). Phytochemical analysis and in vitro biological activity of three Hypericum species from the Canary Islands (Hypericum reflexum, Hypericum canariense and Hypericum grandifolium). Fitoterapia.

[48] Papoulias E., Siomos A.S., Koukounaras A., Gerasopoulos D., Kazakis E. (2009). Effects of genetic, pre-and post-harvest factors on phenolic content and antioxidant capacity of white asparagus spears. Int. J. Mol. Sci..

[49] Gimeno E., Castellote A., Lamuela-Raventós R., De la Torre M., López-Sabater M. (2002). The effects of harvest and extraction methods on the antioxidant content (phenolics, α-tocopherol, and β-carotene) in virgin olive oil. Food Chem..

[50] Bilgin M., Şahin S. (2013). Effects of geographical origin and extraction methods on total phenolic yield of olive tree (Olea europaea) leaves. J. Taiwan Inst. Chem. Eng..

[51] Iqbal S., Bhanger M. (2006). Effect of season and production location on antioxidant activity of Moringa oleifera leaves grown in Pakistan. J. Food Compos. Anal..

[52] Orphanides A., Goulas V., Gekas V. (2013). Effect of drying method on the phenolic content and antioxidant capacity of spearmint. Czech J. Food Sci..

[53] Hossain M., Barry-Ryan C., Martin-Diana A.B., Brunton N. (2010). Effect of drying method on the antioxidant capacity of six Lamiaceae herbs. Food Chem..

[54] El-Hawary S.S., Ashour R.M.S., El-Gayed S.H., Gad H.A., Jaleel G.A.A., El Gedaily R.A. (2020). Genetic, chemical, and biological diversity in Mangifera indica L. cultivars. Pharmacogn. Res.

