# Supplementary material for: Evaluation of Structurally Different Ionic Liquid-Based Surfactants in a Green Microwave-Assisted Extraction for the Flavonoids Profile Determination of Mangifera sp. and Passiflora sp. Leaves from Canary Islands
Source: Molecules. 2020 Oct 15;25(20):4734. doi: 10.3390/molecules25204734 (PMC7594028; doi:10.3390/molecules25204734)
Supplement: Supplementary file 1 [file molecules-25-04734-s001.pdf]

SUPPLEMENTARY MATERIAL

Evaluation of structurally different ionic liquid-based surfactants in a green microwave-assisted extraction for the flavonoids profile determination of *Mangifera sp.* and *Passiflora sp.* leaves from Canary Islands

Kristýna Moučková[1,2], Idaira Pacheco-Fernández[2,3,*], Juan H. Ayala[2], Petra Bajerová[1], Verónica Pino[2,3,*]

[1]*Department of Analytical chemistry, Faculty of Chemical Technology, University of Pardubice, Studentská 573, 53210 Pardubice, Czech Republic*
[2]*Laboratorio de Materiales para Análisis Químicos (MAT4LL), Departamento de Química, Unidad Departamental de Química Analítica, Universidad de La Laguna (ULL), Tenerife, 38206, Spain*
[3]*Instituto Universitario de Enfermedades Tropicales y Salud Pública de Canarias, Universidad de La Laguna (ULL), Tenerife, 38206, Spain*

Table of Contents

<u>Figures</u>

Figure S1 .... page S2

Figure S2 .... page S3

Figure S3 .... page S4

<u>Tables</u>

Table S1 .... page S5

Table S2 .... page S6

Table S3 .... page S7

Table S4 .... page S8

Table S5 .... page S9

Table S6 .... page S10

Table S7 .... page S11

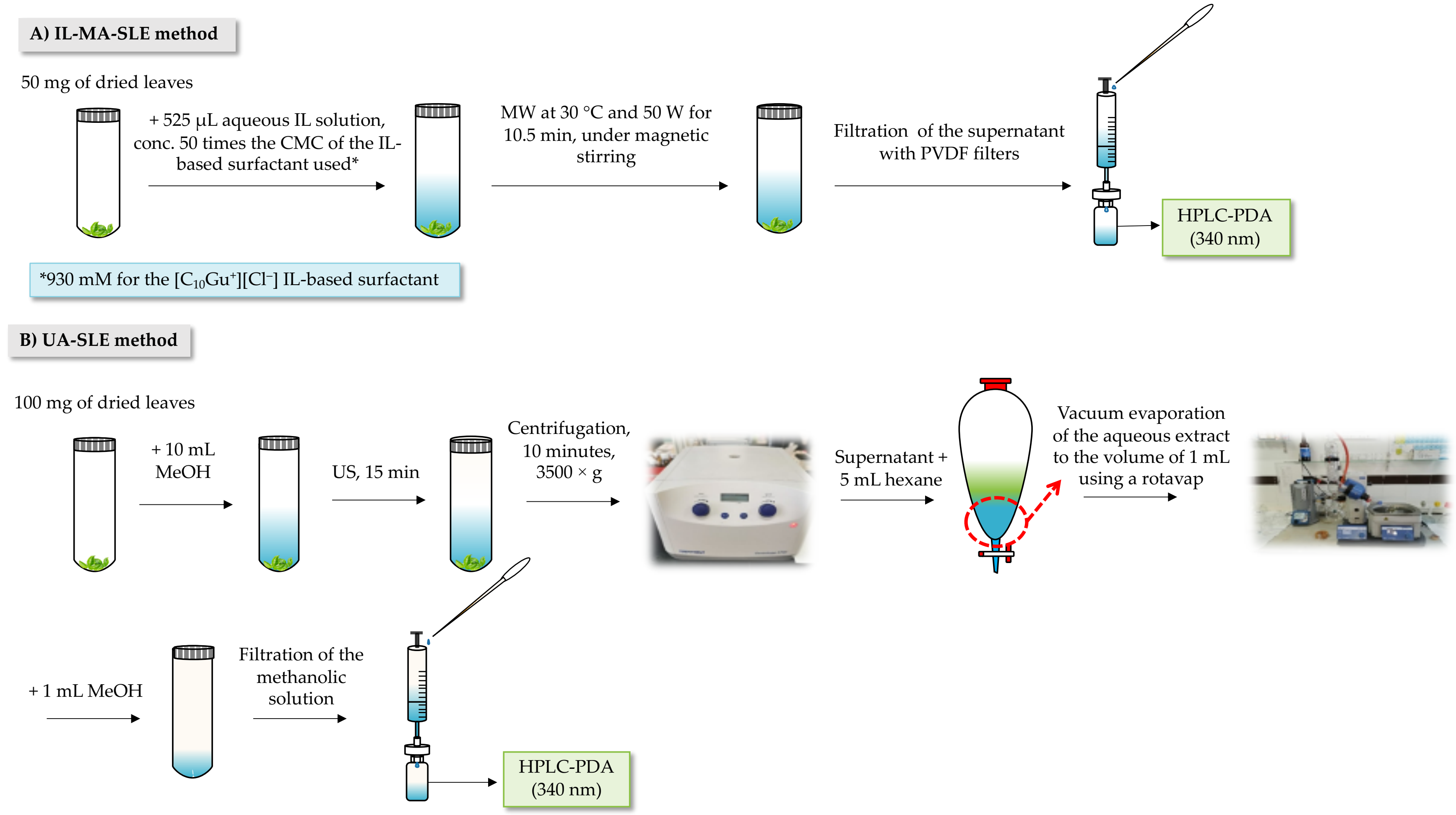

Figure S1. Scheme of A) the proposed IL-MA-SLE-HPLC-PDA method, when performed under optimum conditions, and B) the conventional UA-SLE-HPLC-PDA method, both used for the extraction of flavonoids from fruit leaves.

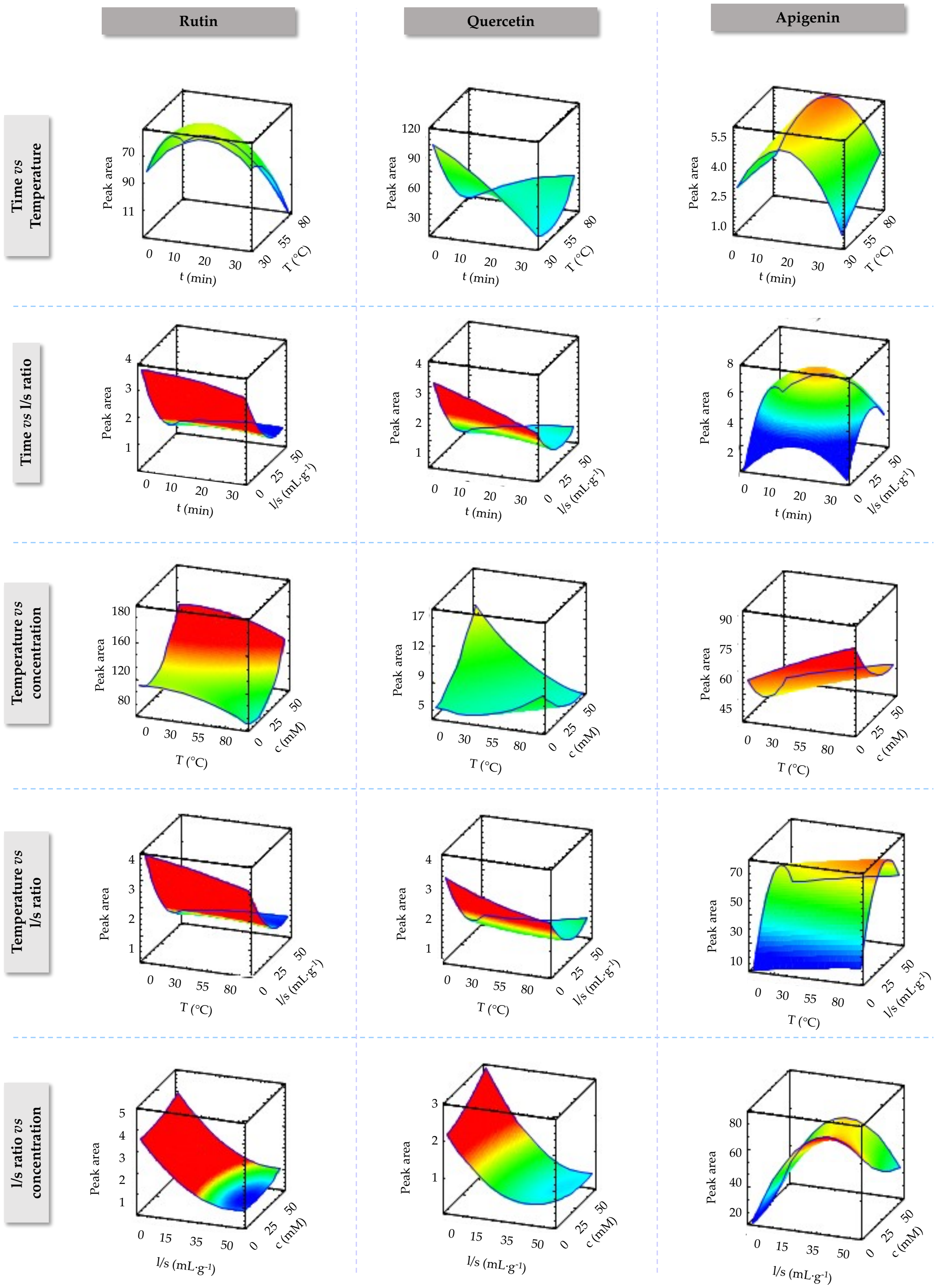

Figure S2. Obtained response surfaces as described by the second order multivariate regression equation for each target flavonoid, presenting the dependency of the peak area with the studied variables.

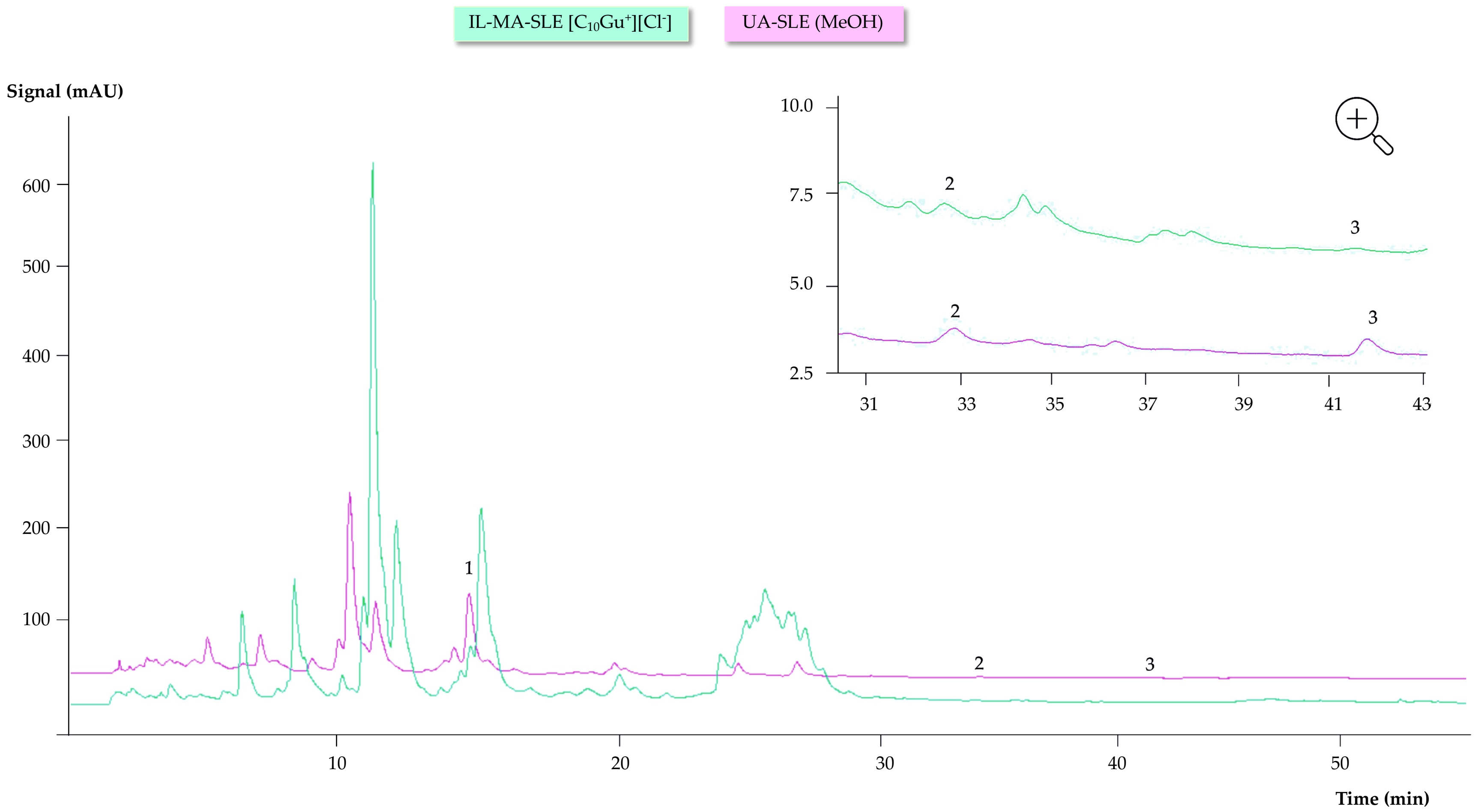

Figure S3. Representative chromatograms obtained for the analysis of *Passiflora sp.* PS032 using the IL-MA-SLE-HPLC-PDA method with the $[C_{10}Gu^{+}][Cl^{-}]$, in comparison with the chromatogram obtained when using the conventional UA-SLE-HPLC-PDA method. Peak 1: rutin, peak 2: quercetin, peak 3: apigenin.

Table S1. Chemical structures and physicochemical properties of the flavonoids determined in this study, obtained from SciFinder® 2020 database.

Analyte	Chemical structure	MW ($g{\cdot}mol^{-1}$)	pK_a	Log K_{ow}[a]
Rutin	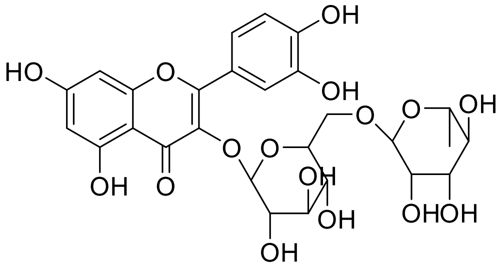	610.52	6.17	-0.90
Quercetin	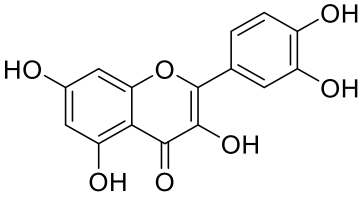	302.24	6.31	1.99
Apigenin	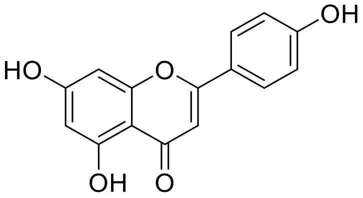	270.24	6.53	2.13

[a] Logarithm of octanol/water partition coefficient at 25 °C.

Tables S2. Matrix of experiments of the Box-Behnken design used for the optimization of the IL-MA-SLE method, including the coded and the operating values.

Run[a]	Coded values[b]				Operating values[b]			
	C_1	C_2	C_3	C_4	β_1	β_2	β_3	β_4
1	-1	-1	0	0	5.00	30.00	30.00	22.95
2	-1	1	0	0	5.00	80.00	30.00	22.95
3	1	-1	0	0	30.00	30.00	30.00	22.95
4	1	1	0	0	30.00	80.00	30.00	22.95
5	0	0	-1	-1	17.50	55.00	10.00	0.90
6	0	0	-1	1	17.50	55.00	10.00	45.00
7	0	0	1	-1	17.50	55.00	50.00	0.90
8	0	0	1	1	17.50	55.00	50.00	45.00
9	-1	0	0	-1	5.00	55.00	30.00	0.90
10	-1	0	0	1	5.00	55.00	30.00	45.00
11	1	0	0	-1	30.00	55.00	30.00	0.90
12	1	0	0	1	30.00	55.00	30.00	45.00
13	0	-1	-1	0	17.50	30.00	10.00	22.95
14	0	-1	1	0	17.50	30.00	50.00	22.95
15	0	1	-1	0	17.50	80.00	10.00	22.95
16	0	1	1	0	17.50	80.00	50.00	22.95
17	-1	0	-1	0	5.00	55.00	10.00	22.95
18	-1	0	1	0	5.00	55.00	50.00	22.95
19	1	0	-1	0	30.00	55.00	10.00	22.95
20	1	0	1	0	30.00	55.00	50.00	22.95
21	0	-1	0	-1	17.50	30.00	30.00	0.90
22	0	-1	0	1	17.50	30.00	30.00	45.00
23	0	1	0	-1	17.50	80.00	30.00	0.90
24	0	1	0	1	17.50	80.00	30.00	45.00
25	0	0	0	0	17.50	55.00	30.00	22.95
26	0	0	0	0	17.50	55.00	30.00	22.95
27	0	0	0	0	17.50	55.00	30.00	22.95

The subscripts refer to the following, 1: extraction time (min); 2: extraction temperature (°C); 3: l/s ratio ($mL \cdot g^{-1}$); and 4: IL-based surfactant concentration (mM).

[a] The number of experiments is given by N = 2k(k–1) + C_0, where k is the number of factors and C_0 is the number of center point repetitions.

[b] The relationship between coded and operational values is given by: $C_i = \frac{\beta_i - \beta_i^0}{\Delta\beta_i} \cdot \alpha$, where C_i is the coded value for the level of factor i, β_i is its real value in an experiment, β_i^0 is the real value at the center of the experimental domain, $\Delta\beta_i$ is the step of variation of the real value, and α is the coded value limit for each factor.

For the extraction time, extraction temperature, l/s ratio and concentration, the minimum values are: 5 min, 30 °C, 10 $mL \cdot g^{-1}$, and 0.9 mM, respectively. The maximum values are 30 min, 80 °C, 50 $mL \cdot g^{-1}$, and 45 mM, respectively. The center points are: 7.5 min, 55 °C, 30 $mL \cdot g^{-1}$, and 2.95 mM, respectively.

Table S3. Obtained values for the constant and coefficients of the second order multivariate regression equation for the fitted response surfaces of rutin, quercetin, and apigenin, as target flavonoids.

**Coefficient**[a]	**Rutin**	**Quercetin**	**Apigenin**
Constant	41687300.00	512030.00	-42617.30
β_1	71028.90	-8404.82	3488.87
β_2	-24583.40	-5079.28	78.75
β_3	-1534080.00	-14381.40	4355.19
β_4	-54115.50	2468.22	41.96
β_{11}	-4968.56	-25.11	-142.89
β_{12}	-1816.22	69.96	18.34
β_{13}	5295.74	133.30	-9.96
β_{14}	-249.25	28.00	-3.22
β_{22}	-1005.11	23.13	-0.99
β_{23}	4287.16	61.99	1.34
β_{24}	69.62	-43.22	-2.73
β_{33}	13203.30	115.11	-59.59
β_{34}	-864.87	-43.37	-22.85
β_{44}	3061.48	20.47	10.45

[a] The subscripts refer to the following, 1: extraction time (min); 2: extraction temperature (°C); 3: l/s ratio ($mL{\cdot}g^{-1}$); and 4: IL-based surfactant concentration (mM), using the IL $[C_{16}C_4Im^+][Br^-]$ as model ionic liquid-based surfactant and *Passiflora flavicarpa* (PS032) as model sample (50 mg).

Table S4. Several parameters obtained from the analysis of the variance (ANOVA) of the experimental results using the BBD.

Variable	Rutin		Quercetin		Apigenin	
	F-Ratio	P-value	F-Ratio	P-value	F-Ratio	P-value
β_1	0.41	0.5364	0.29	0.5997	0.82	0.3841
β_2	0.89	0.3647	0.36	0.5587	0.31	0.5899
β_3	79.11	0.0000	8.78	0.0118	0.07	0.8029
β_4	1.84	0.2000	0.07	0.7971	0.45	0.5142
β_{11}	0.28	0.6049	0.02	0.8894	1.51	0.2432
β_{12}	0.11	0.7424	0.47	0.506	0.07	0.7896
β_{13}	0.62	0.4479	1.09	0.3166	0.01	0.9076
β_{14}	0.00	0.9682	0.06	0.8129	0.00	0.967
β_{22}	0.18	0.6749	0.27	0.6102	0.00	0.9735
β_{23}	1.61	0.2280	0.94	0.3503	0.00	0.9752
β_{24}	0.00	0.9822	0.56	0.4694	0.01	0.9442
β_{33}	13.06	0.0036	2.78	0.1213	1.72	0.2146
β_{34}	0.05	0.8250	0.36	0.5598	0.23	0.6401
β_{44}	1.04	0.3285	0.13	0.7249	0.08	0.7846
R^2	0.896		0.577		0.318	

The subscripts refer to the following, 1: extraction time (min); 2: extraction temperature (°C); 3: l/s ratio (mL·g^{-1}); and 4: IL-based surfactant concentration (mM), using the IL [$C_{16}C_4Im^+$][Br^-] as model IL-based surfactant and *Passiflora flavicarpa* (PS032) as model sample (50 mg).

* Determination coefficient of the quadratic regression.

Table S5. Optimum values obtained with the BBD for each target flavonoid when using the IL-MA-SLE method with the $[C_{16}C_4Im^+][Br^-]$ IL-based surfactant, and *Passiflora flavicarpa* (PS032) as model sample (50 mg).

Analyte	Time (min)	Temperature (°C)	l/s (mL·g^{-1})	IL concentration (mM)
RU	5.0	31.7	10.2	45.0
QU	10.5	30.0	10.5	45.0
AP	15.9	80.0	35.7	0.9

Table S6. Several analytical quality parameters of the HPLC-PDA method for the determination of rutin, quercetin and apigenin.

**Analyte**	**Calibration range (mg·L^{-1})**	**(Slope ± t_{n-2}·SD[a])·10^{-4}**	**R^{2}[b]**	**LOD[c] (µg·L^{-1})**	**LOQ[d] (µg·L^{-1})**	**Intra-day RSD[e] (%)**	**Inter-day RSD[f](%)**
Rutin	0.1 – 500	9.1 ± 0.2	0.9992	40	100	2.32	3.22
Quercetin	0.05 – 150	14.3 ± 0.4	0.9988	20	50	2.37	2.33
Apigenin	0.03 – 150	26.8 ± 0.6	0.9991	10	30	2.08	2.55

[a] Confidence limits of the slope for 10 calibration levels and a confidence level of 95% within the calibration range.

[b] Determination coefficient.

[c] Limit of detection, experimentally determined by decreasing the concentration of the injected standards until a S/N ratio of 3 was obtained.

[d] Limit of quantification, estimated as 10/3 times the LOD, and experimentally verified by the injection of standards at the predicted concentrations.

[e] Relative standard deviation for injections in the same day (n = 3) using a standard concentration of 30 mg·L^{-1}.

[f] Relative standard deviation for injections in three non-consecutive days (n = 9) using a standard concentration of 50 mg·L^{-1}.

Table S7. *Passiflora sp.* and *Mangifera sp.* leaves analyzed in this study for the quantification of flavonoids.

Fruit name	Leaf anatomy	Fruit name	Leaf anatomy
Passiflora sp.		***Mangifera sp.***	
PS032	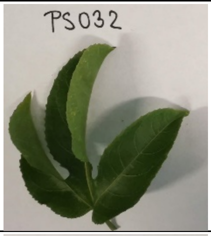	Gomera 1	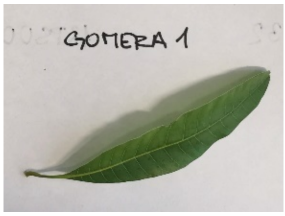
17PS009	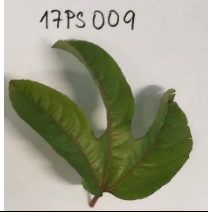	Gomera 3	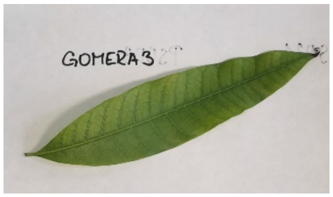
PS003	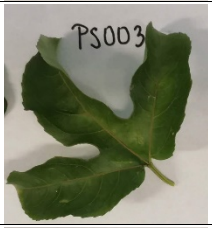	Sweet tart	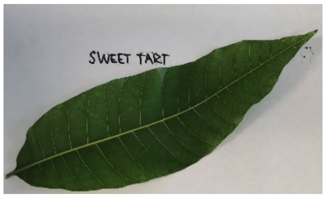
17PS008	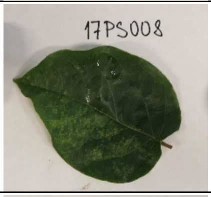	Mun	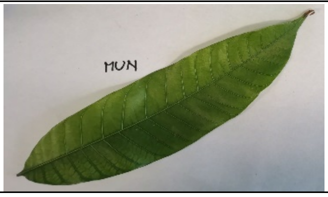
18PS003	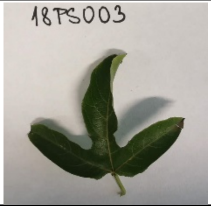		
